# Remotely sensed thermal decay rate: an index for vegetation monitoring

**DOI:** 10.1038/s41598-020-66193-5

**Published:** 2020-06-17

**Authors:** S. S. Kumar, L. Prihodko, B. M. Lind, J. Anchang, W. Ji, C. W. Ross, M. N. Kahiu, N. M. Velpuri, N. P. Hanan

**Affiliations:** 1 ASRC Federal Data Solutions contractor to U.S. Geological Survey (USGS), Earth Resources Observation and Science (EROS) Center, Sioux Falls, SD SD 57198 USA; 2 Department of Plant and Environmental Sciences, New Mexico State University, Las Cruces, NM 88003 USA; 3 Department of Animal and Range Sciences, New Mexico State University, Las Cruces, NM 88003 USA; 4 Department of Plant and Environmental Sciences, New Mexico State University, Las Cruces, NM 88003 USA; 5 International Livestock Research Institute (ILRI), PO Box 30709, Nairobi, 00100 Kenya; 6 International Water Management Institute, 127 Sunil Mawatha, Pelawatte, Battaramulla, Colombo, Sri Lanka

**Keywords:** Environmental sciences, Carbon cycle

## Abstract

Vegetation buffers local diurnal land surface temperatures, however, this effect has found limited applications for remote vegetation characterization. In this work, we parameterize diurnal temperature variations as the thermal decay rate derived by using satellite daytime and nighttime land surface temperatures and modeled using Newton’s law of cooling. The relationship between the thermal decay rate and vegetation depends on many factors including vegetation type, size, water content, location, and local conditions. The theoretical relationships are elucidated, and empirical relationships are presented. Results show that the decay rate summarizes both vegetation structure and function and exhibits a high correlation with other established vegetation-related observations. As proof of concept, we interpret 15-year spatially explicit trends in the annual thermal decay rates over Africa and discuss results. Given recent increases in availability of finer spatial resolution satellite thermal measurements, the thermal decay rate may be a useful index for monitoring vegetation.

## Introduction

Remote sensing has proven to be an invaluable tool for monitoring global vegetation over the last few decades, providing a variety of quantitative measures, retrieved using observations across wavelengths. In broad terms, the visible (VIS 0.4–0.7 μm) wavelengths respond to photosynthetic and non-photosynthetic pigments^1^, the Near Infrared (NIR 0.7–1.4 μm) wavelengths respond to the cellular structure and exhibit Solar Induced Florescence (SIF)^2,3^, and the Short Wave Infrared (SWIR 1.4–3 μm) wavelengths respond to senescent non-photosynthetic vegetation^4^. Further, the anisotropic behavior of vegetation at VIS- SWIR reflective wavelengths have been parameterized to describe vegetation structure^5^. Active sensors (for example light detection and ranging: lidar) using NIR wavelengths have also been used for quantifying vegetation-related structural parameters^6–8^. Beyond NIR/SWIR wavelengths, observations in the microwave (1 cm^−1^ m)^9–11^ region that respond to vegetation water content and structure have also been used for characterization. Observations in different spectral wavelengths provide unique quantitative descriptions on different aspects of vegetation and are being used extensively for remote vegetation monitoring.

The land surface temperature (LST), which can be remotely retrieved using Thermal Infrared (TIR ~10μm) observations over terrestrial systems, is an indicator of the interaction between the vegetation and its local environment^12–16^. Reduction of LST with increasing vegetation cover is well established^15,17^. LSTs have been shown to be lower in pristine forests as compared to secondary growth^18^ and have also been shown to vary by vegetation type^13,19^. The difference between the maximum and minimum diurnal temperatures or the diurnal temperature range (DTR) has been related to biomass heat storage^20,21^.While many studies have used the temporal evolution of LST to characterize land by its thermal inertia^22^ and to diagnose surface energy and water balance^23,24^, few have used thermal information to characterize vegetation^13,17,18^ or biomass^25,26^.

In this work, we build on previous research and capitalize on a cooling curve paradigm to estimate the thermal decay rate that effectively captures land surface thermal dynamics mediated by vegetation. Daytime heating is predominantly radiative (from the sun) while cooling by live vegetation is governed by conductive and convective mechanisms of heat transfer including evapotranspiration (ET) for which Newton’s law^27^ is applicable. The exponential component of Newton’s law, also known as the thermal decay rate constant (${R}_{dk}$ [s^−1^]), is a function of the thermal properties that govern the heat transfer between an object and its surroundings and is inversely related to the density, specific heat, and volume to area ratio^27^. Thus, denser stands of vegetation, with higher specific heat and higher volume to area ratio are expected to exhibit smaller thermal decay rates. Conversely sparser vegetation with lower density, lower specific heat and lower volume to area ratio is expected to have larger thermal decay rates. Along with this dependency on vegetation structure, the decay constant also represents vegetation function as it is also governed by evaporative cooling caused by both evaporation and transpiration^28^ by the living plant tissues and its neighboring surfaces. Thus, the thermal decay rate can be expected to summarize both vegetation structure and function and hence is a novel index for vegetation monitoring.

The theory, assumptions, limitations, and scope of this vegetation-related parameter are presented and discussed herein. Correlations between annual average thermal decay rate constant $\overline{{R}_{dk}}$ and a range of vegetation-related parameters including Normalized Difference Vegetation Index (NDVI), Enhanced Vegetation Index (EVI), Woody percent cover (Woody), Vegetation Continuous Field (VCF) percent tree cover, Leaf Area Index (LAI: Woody and Herbaceous), Vegetation Optical Depth (VOD), SIF, Canopy Height, Above Ground Biomass (AGB), and (ET) and precipitation (Long-term Mean Annual Precipitation (LMAP) and annual precipitation (Precip)) are presented and discussed. These variables were selected because they represent a wide range of vegetation characteristics. The vegetation indices are a measure of greenness while woody percent cover and percent tree cover are the fractional vegetation cover. LAI is a dimensionless variable, defined as the one-sided area of green leaves (m^2^) per unit ground area (m^2^). VOD describes vegetation attenuation properties in microwave wavelengths attributed to the water content in vegetation. SIF is known to be correlated directly to photosynthesis, and canopy height and biomass are physical attributes of vegetation structure. Precipitation is also included in this work as it is one of the fundamental drivers of vegetation. Inter-correlation among these parameters are expected because they are all related to vegetation structure and function. Results from a comprehensive cross-correlation study are presented with a proof of concept time-series application for sub-Saharan Africa. Results suggest that the thermal decay rate relationship to vegetation biomass has potential as a new index for vegetation monitoring and modelling.

## Results

**Figure 1:**
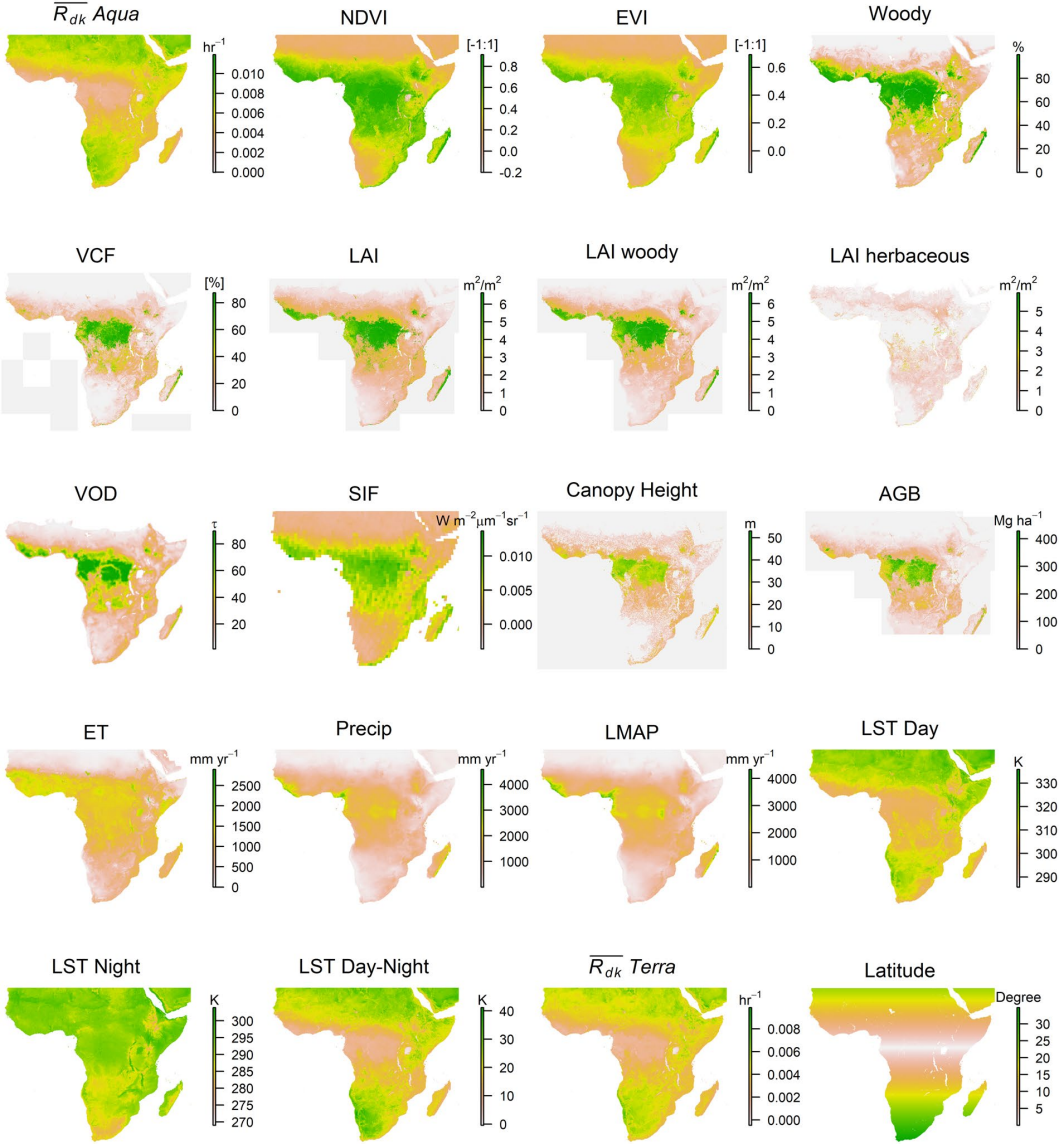
Maps of all variables used in this work for the sub-Saharan Africa study area. Greener shades represent higher values while browner tones represent lower values.

The relationship between ($\overline{{R}_{dk}}$) and vegetation-related parameters is complex because it depends on many factors including vegetation type, density and structure, water content of both vegetation and soil, thermal properties of land surface components and seasonal weather. Here we evaluate $\overline{{R}_{dk}}$ through comparison to a suite of remotely sensed variables related to vegetation structure and function (see Materials and Methods).The spatially explicit data used for this work are illustrated in Fig. 1, including $\overline{{R}_{dk}}$ maps from both the National Aeronautics and Space Administration (NASA) Aqua and Terra Earth-observing satellites.

### Sensitivity to temporal sampling

**Figure 2:**
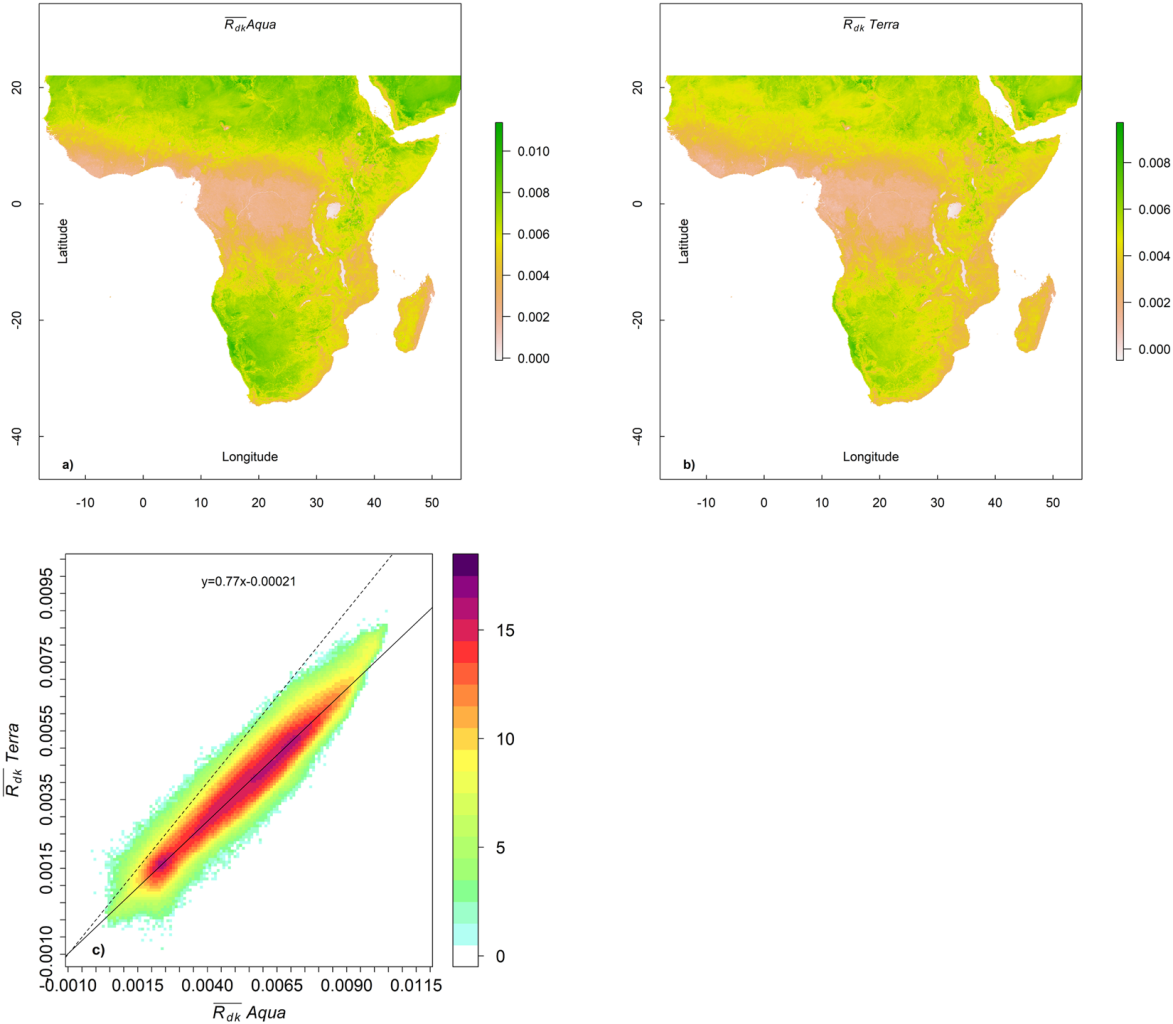
$\overline{{R}_{dk}}$ values for 2005 for the sub-Saharan Africa study area derived using MODIS Aqua only (**a**) and MODIS Terra only (**b**). The relationship between the $\overline{{R}_{dk}}$ values derived using Aqua only and Terra only is also shown (**c**). Density is displayed using a (2^n^ − 1) scale, with *n* shown in the legend. The dashed line marks the 1: 1 line and the solid line is from RMA regression coefficients. Larger values are expected for Aqua as its time of over pass coincides with the time of maximum diurnal differences. The MODIS Terra over pass happens earlier when the diurnal temperature differences are not near their maximum. The Spearman’s and Pearson’s *r* is >0.97.

Spatially explicit 2005 $\overline{\,{R}_{dk}}$ values derived using Moderate Resolution Imaging Spectroradiometer (MODIS) Aqua are compared with 2005 $\overline{{R}_{dk}}$ values derived using MODIS Terra in Fig. 2. The $\overline{{R}_{dk}}$ values derived using Aqua are expected to be higher as the over pass times of the Aqua satellite occurs at ~1:30 PM and ~1:30 AM near the equator^29^, closer to the times of diurnal maximum and minimum temperatures^30^. The over pass times of Terra occur earlier as compared to Aqua at 10:30 AM and 10:30 PM near the equator^29^. Despite this time difference, a strong linear relationship between the two derivations of $\overline{{R}_{dk}}$ is observed (*r* > 0.97 Fig. 2). The reduced major access regression (RMA) coefficients suggest that the slope is ~ 0.77 while the intercept is almost negligible (<~ 10% of the low values;). As a result, the $\overline{{R}_{dk}}\,$values derived using Aqua are used for all further analyses presented in this work.

### Relationship to biotic variables

**Figure 3:**
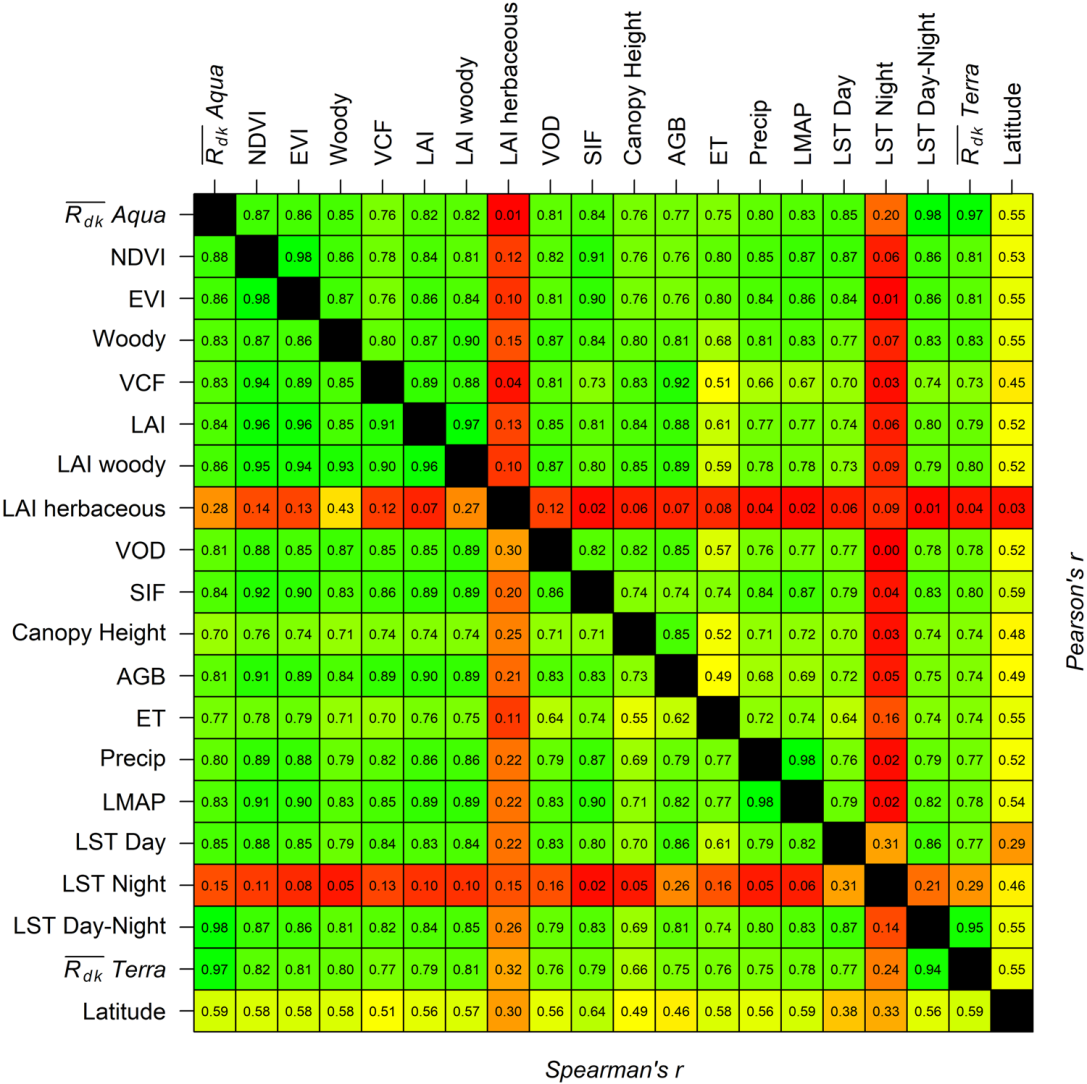
Color-coded cross-correlation values between the different variables analyzed in this work. Locations with less than 100 mm of MAP were excluded from this study. Shades of red, yellow and green represent increasing magnitude of correlations irrespective of the sign. Absolute values of latitude were used for estimating correlation. Both Pearson’s (above the solid black diagonal line) and Spearman’s (below the diagonal line) correlation values (*r)* are presented. See Table 1 for variable description.

**Figure 4:**
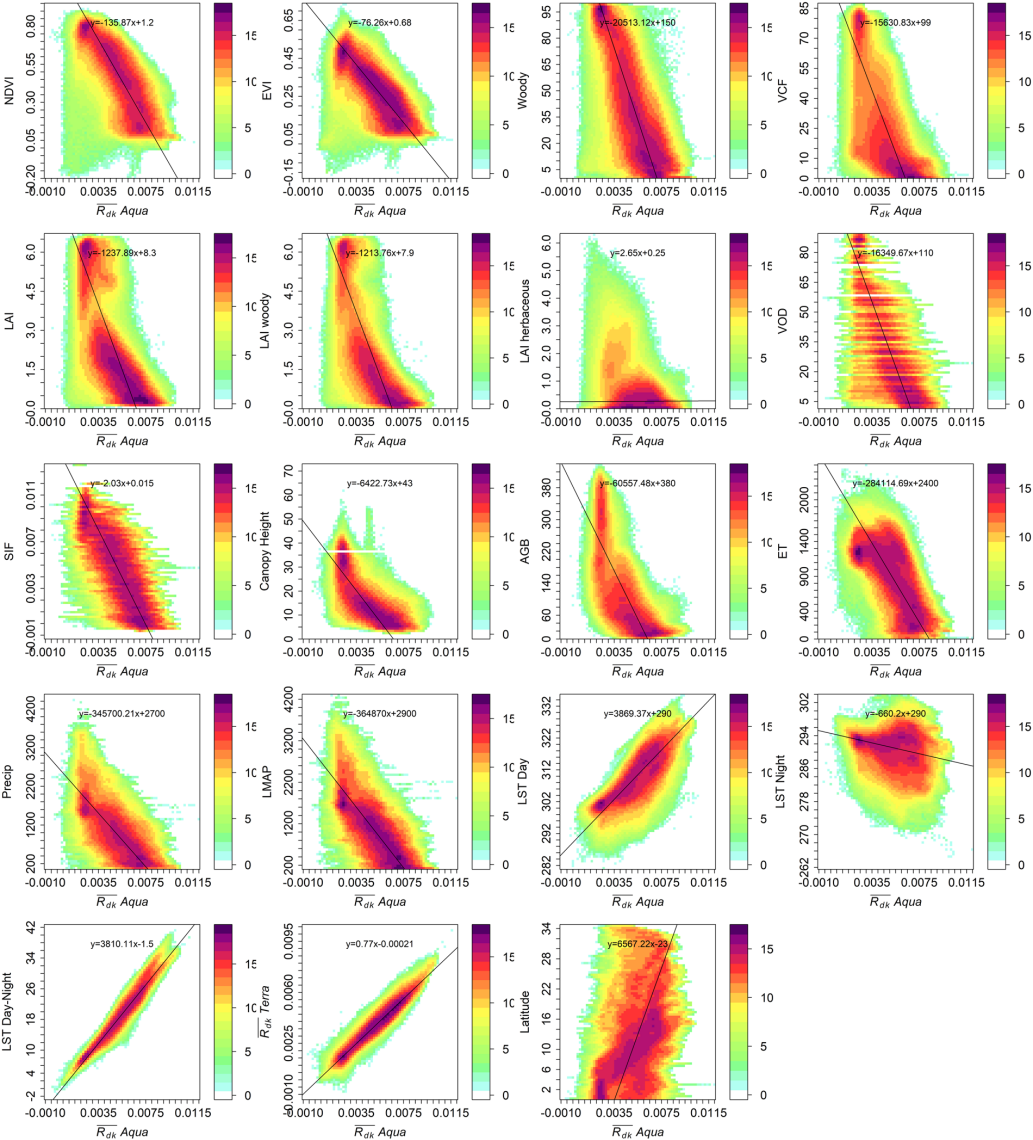
Scatter plots showing the relationship of $\overline{{R}_{dk}}$ to all other variables considered in this study. Density is color coded on a 2^n^ − 1 scale.

Variables relating to the vegetation structure and function are considered biotic for the purpose of this study. The inter-variable Pearson’s and Spearman’s correlation *r* is summarized in a color-coded table (Fig. 3) and individual scatter plots in Fig. 4 illustrate the relations to $\overline{{R}_{dk}}$ values. Only locations with long-term mean annual rainfall of >100 mm/year are considered in this correlation analysis. We tabulate both correlation indices because Pearson’s *r* evaluates the degree of linear relationships, while the Spearman’s *r* provides a measure for monotonic relationships, which may or may not necessarily be linear. In general, we found the Spearman’s correlations to be similar to and sometimes marginally higher than the Pearson’s correlation, suggesting more monotonic than strictly linear relationships. This is also evident from Fig. 4. The $\overline{{R}_{dk}}$ values are highly and linearly correlated with the common vegetation indices NDVI and EVI (~0.86+). Similar high correlations were observed with woody LAI estimates (0.86; Fig. 3), VOD (~0.8) and SIF (0.84), but $\overline{{R}_{dk}}$ is poorly correlated with herbaceous LAI (0.28) among the biotic variables. The highest inter variable correlation of over 0.98 was observed between the NDVI and EVI among the biotic variables, which is expected as both were derived from the same sensor and similar spectral bands. It must be noted that poor inter variables correlations at continental scales may have been impacted by differences in the temporal span of the variable used. This is especially true for LAI and Woody/Herbaceous LAI wherein the available 2008 data were used in this work. However, this temporal mismatch is less of an issue with correlation of the $\overline{{R}_{dk}}$ values with other variables because they were compared for similar time period as noted in the data section. In general, $\overline{{R}_{dk}}$ values showed correlation values >0.76 (Pearson’s *r*) for most of the vegetation-related variables and are broadly similar to correlations observed amongst the vegetation-related variables.

The DTR, defined here as the LST difference between day and night (LST _day-night_), is known to be related to the biomass heat storage and has been related to the vegetation cover^20,21^. The corroborating results shown in Fig. 3 indicate a high correlation of annual average (LST _day-night_) with biotic parameters. Interestingly, both Spearman’s and Pearson’s correlations of the $\overline{{R}_{dk}}$ is typically higher, although marginally so (~ 0.03), than the correlation of (LST _day-night_) for almost all (except EVI, which is equal) vegetation-related parameters. The higher correlation with vegetation-related parameters implies that while (LST _day-night_) is correlated to biotic variables, the $\overline{{R}_{dk}}$ parameterization of LST is more closely related.

### Relationship with abiotic variables

As expected, the $\overline{{R}_{dk}}$ values are highly correlated (Fig. 3) to the LST_day_, ET and LMAP. In general, these correlation values are similar to the correlation of other established vegetation- related parameters with these abiotic variables (Fig. 3). Similar to observations with variables related to vegetation structure and function, the Spearman’s correlations are similar and marginally higher than the Pearson’s correlation for the same variable pair, and suggest the relationships are monotonic and not necessarily linear as seen in Fig. 4. As expected, the correlation is higher (0.83; Pearson’s) with the LMAP than for the 2005 annual Precip (0.80), because long-term precipitation^31^ is one of the drivers of woody vegetation presence/absence. These results clearly indicate that the $\overline{{R}_{dk}}$ is more closely related to biotic variables than to abiotic variables and thus has potential as a novel vegetation index.

### Spatially explicit time series analysis

**Figure 5:**
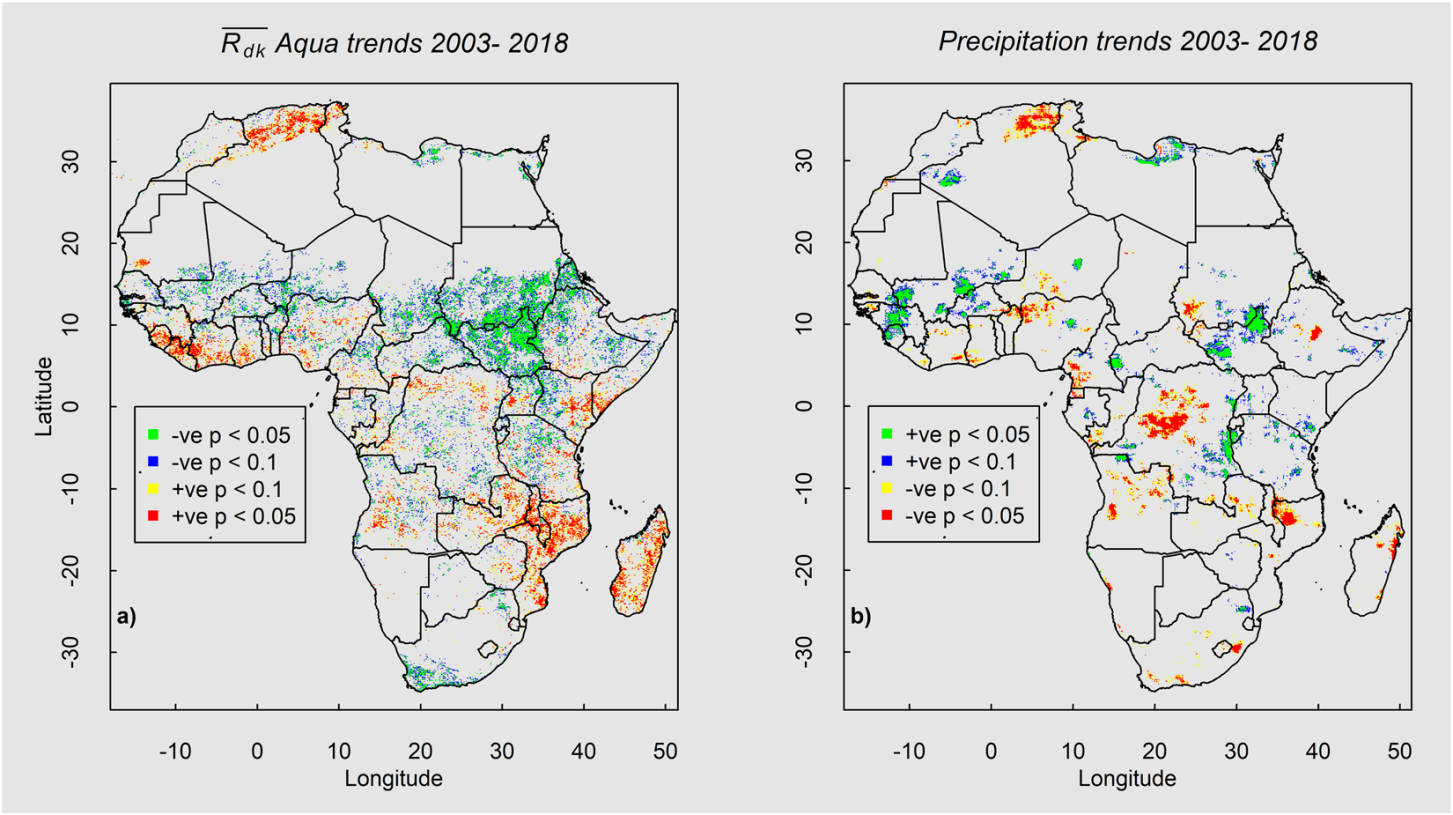
Spatial patterns of decreasing (blues and greens) and increasing (yellows and reds) trends in (**a**) $\overline{{R}_{dk}}$ and (**b**) decreasing (yellows and reds) and increasing (blues and greens) precipitation, colored by their statistical significance (see legend). Vegetation trends are interpreted as the inverse trend in $\overline{{R}_{dk}}$ values, with negative trends indicating increase in biomass. Results illustrated are restricted to only those regions that had over 100 mm yr^−1^ of MAP.

Time series of $\overline{{R}_{dk}}$ may help identify trends over regions experiencing change in vegetation structure and/or function (e.g. growth, deforestation/reforestation, loss and recovery of vegetation following disturbance (drought, flood, fire, etc.) or vegetation change relating to vegetation community change (shrub encroachment or invasion of exotic species)). Figure 5 shows the spatial distribution of significant increasing or decreasing trends in $\overline{{R}_{dk}}$ that could indicate such changes (a) over the study area and (b) a similar analysis with annual precipitation for reference. The results span a 15-year time period from 2003–2017. High and moderate statistical significance (*p* value < 0.1 and <0.05) is inferred from Mann Kendal tests^32^ for the $\overline{{R}_{dk}}$ and precipitation values paired with years of observation (2003–2017). An increasing trend in the $\overline{{R}_{dk}}$ values is interpreted as a decreasing trend in the vegetation (e.g. loss of woody vegetation) and is shown as red tones in Fig. 5a.

Increasing trends in vegetation (i.e. gain of woody vegetation) are evident in the northern and eastern regions of Africa while decreasing trends are seen in the southern and western regions. The above-described increasing and decreasing patterns of vegetation are broadly similar to patterns inferred by previous studies^33^ using VOD data between 1992 and 2011, and between 2010 and 2016^34^, studies using VIS-SWIR^35^ data between 2000 and 2015 for the whole of sub-Saharan Africa and over the west African Sahel^36,37^. Past work^38,39^ has shown with field observations and from remote sensing data that the Sahel region south of the Sahara Desert has been experiencing vegetation “re-*greening*”, particularly relating to recovery of woody populations. This trend for the Sahel is also seen in the $\overline{{{\boldsymbol{R}}}_{{\boldsymbol{dk}}}}$ trends (Fig. 5a; ~10^0^ to 20^0^ Lat, −15^0^ to 30^0^ Lon). Our results suggest that, although there are regions experiencing green-up and increase in precipitation, not all regions experiencing green-up in the Sahel show a significant increase in precipitation between 2003 and 2015. In the west, a distinct degrading trend in vegetation is evident in Liberia (Fig. 5a; ~ 5^0^ to 10^0^ Lat, −15^0^ to 5^0^ Lon) with no significant decrease in precipitation for the same time period (Fig. 5b). This decreasing trend may be attributed to shifting land use from forest to agricultural lands observed by past land cover studies^36^ over regions of west Africa. A distinct and large area of significant increasing trends in vegetation and in precipitation is evident in the east in the upper Nile Basin of Sudan (the Sudan; Fig. 5a; ~10^0^ Lat, 35^0^ Lon). Recent studies^40^ have suggested a 14% increase in vegetated wetlands in this region for the 1999 to 2006 time period. Differences between the $\overline{{{\boldsymbol{R}}}_{{\boldsymbol{dk}}}}$ and precipitation trends at local scale are expected due to coarser-scale precipitation data compared to the land surface data as well as local edaphic factors that also mediate vegetation^31,41^. For example, the dense forest in central Africa has been experiencing a significant decline in precipitation, however, a similar decline in the vegetation is only seen in scattered regions. These observations indicate that changes in vegetation as inferred from $\,\overline{{{\boldsymbol{R}}}_{{\boldsymbol{dk}}}}$ are more likely driven primarily by vegetation change, rather than changing precipitation, and thus ${{\boldsymbol{R}}}_{{\boldsymbol{dk}}}\,$has potential as a vegetation index.

## Discussion

Remote observations in the thermal bands using Earth-observing satellites have been known to be useful to characterize land cover and, in this work, we show how the diurnal change in temperatures could be parameterized and interpreted to characterize vegetation structure and function. We show that the thermal decay rate (${R}_{dk}$), derived using principles governing the rate of cooling under certain assumptions, provides an index of vegetation with theoretical and empirical justification. We show that the annual average $\,\overline{{R}_{dk}}$ has interesting properties suitable for vegetation monitoring and modeling. In addition, the relationship of $\overline{{R}_{dk}}$ to biomass heat storage may be useful as a proxy in land surface models to improve energy balance^21^ calculations.

As expected, thicker dense vegetation had smaller decay rate values, while sparser vegetation was shown to have higher decay rate values. Spatially explicit time series analysis of the $\overline{{R}_{dk}}$ values and precipitation showed spatial agreement with known regions of vegetation green-up and degradation over Africa in the last decade^33^.

The derivation of $\overline{{R}_{dk}}$ can be applied to existing satellites such as the Visible Infrared Imaging Radiometer Suite (VIIRS)^42^ and the Sentinel 3A and 3B^43^ satellites with due consideration of their specific over-pass times. The decreased spatial variability in the nighttime temperatures (Fig. 1) opens the possibility of fusing available finer daytime satellite land surface temperatures with coarser resolution nighttime LST observations to derive finer spatial resolution $\overline{{R}_{dk}}$ . The successful launch and commissioning of the **ECO**system **S**paceborne **T**hermal **R**adiometer **E**xperiment on **S**pace **S**tation (ECOSTRESS; https://ecostress.jpl.nasa.gov/) mission, with improved thermal band spatial resolution, increases the number of satellites with thermal remote sensing capability (VIIRS, Sentinel 3A and B) and can be expected to pave the way for operationally using thermal bands for monitoring vegetation globally. Thus, future remote sensing programs can also consider using $\overline{{R}_{dk}}$ retrievals for vegetation monitoring.

## Methods

### Study area

Sub-Saharan Africa was chosen for this study because it presents a wide range of vegetation conditions, from desert to savannas and moist tropical forests, in both southern and northern latitudes, making it a suitable study area to evaluate the relationship between *R*_*dk*_ and other biotic and abiotic parameters.

### Data and processing

Table 1 lists all data used in this work and includes key attributes, processing applied and reference to data products/source. All spatially explicit data used in this work are remotely sensed data that are freely available, either via data archives or direct from the authors. The variables used in this work are broadly categorized as biotic (vegetation related) and abiotic for convenience and clarity of this paper. Cloudy and missing data were excluded while collating the datasets. All data variables are directly available as data layers or computed as described in the column labeled processing in Table 1. Spatial datasets were mapped to a common spatial resolution of 1 km. The nearest neighbor method was used for downscaling data from a coarser spatial scale to finer scale, while regional mean was computed for upscaling data. All image pre/processing, subsequent analysis including graphical illustration of results were undertaken using Raster^44^ and RGDAL^45^ packages in the R open source environment^46^.

**Table 1:** Data description and source.

	Variable	Variable acronyms [units]	Satellite product name	Spectral bands	Spatial resolution	Temporal resolution	Processing	Temporal coverage	Source
Biotic	Vegetation Indices	NDVI, EVI [dimless]	MOD13Q1	VIS-NIR	250 m	8 day	Annual average and aggregation (mean) to 1 km	2005	^47^
	Vegetation Continuous Fields	VCF [% Tree cover]	MOD44B	VIS-NIR	250 m	Annual	Aggregation (mean) to 1 km	2005	^48^
	Sub-Saharan Woody	Woody [% Woody cover]	N/A	VIS-NIR, microwave	1 km	Annual	None	2005	At the time of publication, data are not publicly available (See data availability)
	Vegetation Optical Depth	VOD [τ dimless]	N/A	microwave	0.25^0^ × 0.25^0^	Annual	Resample (nearest neighbor) to 1 km	1992–2011	^33^
	Canopy height	Canopy Height [m]	N/A	LiDAR	1 km	Annual	Resample (nearest neighbor) to 1 km	2005	^49^
	Above Ground Biomass	AGB [Mg ha^−1^]	N/A	VIS-NIR, LiDAR	1 km	Annual	Resample (nearest neighbor) to 1 km	2005	^50^
	Leaf Area Index	LAI, LAI Woody, LAI Herbaceous [m^2^ m^−2^]	MOD15A2, LAI Woody/Herbaceous	VIS-NIR	1 km	Annual	N/A	2008	^51,52^
	Solar Induced Fluorescence	SIF [W m^−2^sr^−1^mm^−1^]	N/A	NIR	2^0^ × 2^0^	monthly	Annual average and resample to 1 km	2015	^53^
Abiotic	Land surface temperature	LST Day, LST Night [K]	MYD11A2	TIR	1 km	8 day	Annual average	2005	^14^
		$\overline{{R}_{dk}}$ Aqua [hr^−1^] $\overline{{R}_{dk}}$ Terra [hr^−1^]	MYD11A2 MOD11A2	TIR	1 km	8 day	Eq. (6) MYD, Equation (6) MOD.	2003–2017	This work
	Precipitation	Precip, LMAP [mm year^−1^]	CHIRPS	TIR	0.05^0^ × 0.05^0^	Monthly	Annual and 30-year average	1981–2011	^54^
	Evapotranspiration	ET [mm year^−1^]	N/A	TIR	1 km	Monthly	Annual average	2005	^55^

### Theoretical considerations

Newton’s law of cooling postulates that the rate of change of temperature of an object is proportional to the temperature difference between the object and its surroundings^27,56,57^. This can be mathematically expressed as:

1 $$\frac{d(T(t))}{dt}=-{R}_{dk}(T(t)-{T}_{a})$$

where *T(t)* [K] is the instantaneous temperature at a given time *t* [s], *T*_*a*_ [K] is the ambient temperature, and ${R}_{dk}$ [s^−1^] is the thermal decay rate. It should be noted that (Eq. 1) makes certain implicit assumptions in its derivation that are discussed in the next section. The solution^27^ to (Eq. 1) is

2 $$T(t)={T}_{a}+({T}_{0-}{T}_{a})\,{e}^{-{R}_{dk}t}$$

where *T*_0_ is the initial temperature at *t* = 0, and (Eq. 2) can be rewritten as:

3 $$\frac{T(t)}{{T}_{o}}={e}^{-{R}_{dk}t}+\frac{{T}_{a}}{{T}_{o}}(1-{e}^{-{R}_{dk}t})$$

The second term in (Eq. 3) is small and can be ignored if we assume that the object is cooling from a relatively higher daytime (*T*_*o*_) temperature to a cooler *T*_*a*_ such that *T*_*a*_ ≪ *T*_*o*_, although this assumption may not be strictly valid at all times and locations (discussed below). Ignoring the second term and rewriting *T*_0_ as the daytime (*T*_*d*_) temperature cooling towards the nighttime temperature (*T*_*n*_) over a time period from 0 to *t* = $\Delta t$ in (Eq. 3) yields:

4 $${R}_{dk}=\,\log \left(\frac{{T}_{d}}{{T}_{n}}\right)/\Delta t$$

Theoretically, ${R}_{dk}$ is related to the intrinsic properties of the object and the nature of its interactions with the environment as:

5 $${R}_{dk}={\alpha }_{tot}\frac{1}{\rho .c.\left(\frac{V}{A}\right)}$$

where ${\alpha }_{tot}$ is the effective equivalent heat transfer coefficient considering all mechanisms of heat transfer (conduction, convection and radiation) to its surroundings, *ρ* [kg m^−3^] is the density, *c* [kg m^2^ k^−1^] is the specific heat and V [m^3^]/A[m^2^] is the volume to surface area of the object^27^.

Over objects with extended spatial spans such as pixels of vegetated regions, ${R}_{dk}$ is the collective representation of the rate of cooling and is governed by many factors including the conductive, convective and radiative transfers of heat between neighbors (neighboring soil, plants and atmosphere). In this scenario, ${R}_{dk}$ also incorporates the effects due to evapotranspiration and heat storage dynamics by individual vegetation components, each with its distinct thermal properties. Thus, as evidenced from (Eq. 5) dense vegetation stands, such as forests with objects (trees) that have high volume to area ratio, high density and high heat capacity, will have smaller ${R}_{dk}$ values. Conversely, sparsely vegetated surfaces with high proportions of bare soil or grass with low density, low specific heat and low volume to area ratio will have larger ${R}_{dk}$ values. When calculating ${R}_{dk}$ over large areas, the presence of water bodies (high specific heat), wetlands, or areas with higher soil moisture will also lower ${R}_{dk}$, confounding the detection of vegetation density to some extent.

In this work, we compute the annual average thermal decay rate using remotely sensed day/night land surface temperature to minimize the effects of seasonality as:

6 $$\overline{{R}_{dk}}={\sum }_{1}^{i}\,\log \left(\frac{{T}_{d}}{{T}_{n}}\right)/\Delta t/i$$

where $\overline{{R}_{dk}}$ represents the annual mean and *i* is the number of paired day/night observations in that year.

### Assumptions and limitations

The validity of Newton’s law and associated assumptions govern the applicability of remotely sensed ${R}_{dk}$. Newton’s law of cooling is valid when heat transfer is largely due to conduction and convection, rather than radiation. If heat transfer is dominated by radiation, then the difference in temperature of the object and its ambient surroundings should be small^27,56,57^ to maintain validity of Newton’s law. Further, it assumes that the LST is homogenous within each pixel and is cooling towards an ambient temperature that is constant and does not change over time.

These assumptions may not be strictly valid when considering the ${R}_{dk}$ of a land surface. Failure of the assumptions may manifest as a bias, which could potentially be constrained and, if small, may be ignored. Further, the temperature of the vegetated surface and its ambient temperature may or may not be uniform or constant, which can also bias the estimation of ${R}_{dk}$ using (Eq. 4). Equation (4) is an approximation with an assumption that *T*_*a*_ ≪ *T*_*o*_ to avoid explicit estimation of the second term in (Eq. 3). However, if it is non-negligible, the second term in (Eq. 3) may introduce bias in the simplistic reduction to (Eq. 4).

These biases may change with location and time depending on local conditions including weather/season, vegetation type, clouds, atmospheric conditions and interaction with neighbors. Further, satellitebased observations are limited to their specific over pass times that may not coincide with the maximum and minimum diurnal temperatures. However, these time- and season-based biases may potentially be constrained by examining the relationship between the $\overline{{R}_{dk}}$ and variables driving the bias at specific locations. Further, formulation of (Eq. 4) does not explicitly model the changing sun angles and/or cloud cover through time. Instead of explicitly modeling these potential biases, we maintained consistency in the time of day of sampled observations to constrain the impact of these potential biases on ${R}_{dk}$. It should be noted that the formulation of (Eq. 4) also does not consider temperatures below freezing, nor does it consider the possibility of active heat sources such as fire. The validity of the assumptions may also be sensitive to the scale of observation and spatial distribution of objects.

## Data Availability

Data used in this work were downloaded from freely available public domains or can be obtained from the respective corresponding authors. MODIS data were downloaded from EARTH DATA https://ladsweb.modaps.eosdis.nasa.gov/. Precipitation and evapotranspiration data were downloaded from U.S. Geological Survey FEWS NET Data Portal https://earlywarning.usgs.gov/fews. The sub-Saharan Woody cover data may be requested from NPH, and the woody/herbaceous LAI data are available from the Dryad Digital Repository, 10.5061/dryad.v5s0j.
